# Longitudinal Association Between Children’s Callous-Unemotional Traits and Social Competence: Child Executive Function and Maternal Warmth as Moderators

**DOI:** 10.3389/fpsyg.2019.00379

**Published:** 2019-02-28

**Authors:** Hyunah Kim, Hyein Chang

**Affiliations:** Department of Psychology, Sungkyunkwan University, Seoul, South Korea

**Keywords:** callous-unemotional (CU) traits, social competence, executive function, maternal warmth, Panel Study on Korean Children

## Abstract

This study examined the longitudinal association between children’s early callous-unemotional (CU) traits and social competence in the transition to school-age, and tested whether this relationship was moderated by child executive function and maternal warmth. Participants were 643 children (49% girls) who were part of the Panel Study on Korean Children (PSKC) of the Korea Institute of Child Care and Education (KICCE). Mothers rated children’s CU at 5 years and executive function at 8 years, and maternal warmth at 5 years. Teachers reported on children’s social competence at 8 years. Results of the model including child executive function as the moderator indicated that deficits in child executive function and child sex (boys) predicted lower social competence. In addition, the moderating effect of executive function on the relationship between CU and social competence approached a trend such that CU predicted lower social competence only for children with lower executive function. In the model that included maternal warmth as a moderator, CU traits was associated with lower social competence, and this effect was more pronounced for boys as indicated by a significant effects of CU × child sex on social competence. The findings are discussed with respect to considering individual and contextual factors by which early CU becomes associated with individual differences in children’s social competence.

## Introduction

Children’s social competence refers to their ability to effectively interact in relational contexts by considering their own as well as others’ perspectives, and utilizing adequate social skills (Rose-Krasnor, 1997). Social competence has been a popular topic for research because it is not only a key aspect of children’s functioning, but also a significant indicator of children’s adaptation in other domains including self-esteem (Walker et al., 2002), externalizing and internalizing psychopathology (Bornstein et al., 2010), and academic achievement (Zhou et al., 2010). As such, identifying risk factors as well as mechanisms to variations in children’s social competence may inform early prevention efforts to promote their healthy development.

Of particular focus in this study was to examine pathways by which children’s early manifestations of callous-unemotional (CU) features may become associated with their social competence at early school-age. Although CU may compromise a child’s interpersonally competent behaviors, earlier studies have typically focused on the relations of CU and conduct problems rather than social competence (Frick et al., 2014). More importantly, few studies have explored intrachild and contextual factors that might exacerbate or buffer potentially harmful effects of early CU on children’s later social competence. Thus, the goals of this study were to investigate the longitudinal association of children’s CU and social competence, and to test whether child executive function and maternal warmth might moderate those processes.

### CU Traits and Social Competence

CU traits encompass aspects of an individual’s pattern of behavior that reflects a lack of empathy, poor affect, and low levels of guilt (Frick, 1994). It should be noted that CU “traits” are also referred as CU “behaviors” particularly in studies with young children whose behaviors may not necessarily indicate stable traits (Hyde et al., 2013). However, our decision to use the term *CU traits* in this study did not reflect a particular belief regarding this issue, but more based on the fact that this term has been more commonly used in the literature compared to other alternatives (e.g., Waller et al., 2012, 2014).

Children who show higher levels of CU may have more difficulty in relational contexts, because the deficits that these children experience are key to successful social interaction (Sallquist et al., 2009; Roos et al., 2014). Specifically, CU traits are characterized by a profound lack of foundational skills such as empathy and perspective taking that are critical for reciprocal relationships and prosocial behaviors (Davis, 1983; Eisenberg et al., 2006). Although CU traits and social competence may emerge seemingly simultaneously from early ages (Howes, 1987; Kimonis et al., 2006), they are distinct, albeit related, constructs in that CU traits is a stable, dispositional factor (Dadds et al., 2005; Obradović et al., 2007) whereas social competence is an index of child outcome that may be more malleable and determined be many contextual and individual factors such as CU traits (Howes, 1987). Additionally, CU traits is primarily characterized by affective deficits whereas social competence encompasses more diverse set of interpersonal skills, some of which may be influenced by individual differences in CU traits.

Additionally, children with CU traits are at increased risk for conduct problems which may lead to peer rejection (Webster-Stratton and Lindsay, 1999; Frick et al., 2003). This may hinder them from experiencing emotional interaction and forming intimate bond with peers. In addition, given that they are insensitive to negative stimuli such as punishment (Pardini, 2006) and less likely to expect negative outcomes from their conduct behaviors (Pardini and Byrd, 2012), they may have difficulty understanding the impact of their aversive behaviors which could deteriorate their social relationship.

Indeed, empirical studies have accumulated evidence that children who demonstrate higher levels of CU also show lower levels of social competence. For instance, a study with elementary school students reported that higher levels of CU traits predicted lower levels of social competence rated by both teacher and children even after controlling for externalizing behavior (Haas et al., 2018). Similarly, in another study, affective aspects of psychopathic traits, which are conceptually similar to CU traits, were associated with peer rejection rated by peers, again even after accounting for the effect of behavior problems (Piatigorsky and Hinshaw, 2004).

Many researchers focused on the role of conduct problems in explaining the association between children’s CU traits and social competence. A robust body of studies have accumulated evidence that CU traits are predictive of more severe behavior problems (Frick et al., 2003, 2005). Given that conduct problems are likely to lead to social incompetent behaviors (Chen et al., 2010), the association of CU traits and social incompetence may be explained by children’s externalizing behaviors. In fact, some studies have demonstrated that CU traits may not be related to social competence after controlling for variations in children’s ADHD and ODD symptoms (Pardini and Fite, 2010).

Nevertheless, many studies have provided evidence that CU traits may have unique predictability on social incompetence that is not accounted for by externalizing behavior. For example, CU traits, but not impulsivity or conduct behaviors, were distinctively associated with foundations of social incompetence such as low empathy and perspective-taking (Pardini et al., 2003; Pardini and Byrd, 2012). In addition, the relations between CU traits and social competence were still significant in earlier investigations that have controlled for levels of child externalizing behavior (Piatigorsky and Hinshaw, 2004; Haas et al., 2018). These results indicate that CU traits are likely to have unique influences on social competence above and beyond conduct problems. Furthermore, not all children with CU traits develop behavior problems (Frick et al., 2005), suggesting that the association of CU traits and social competence cannot be fully explained by conduct behaviors.

However, there is a general dearth of research on the association of CU traits and social competence, and the existent literature also suffers from a few limitations. First, many studies have examined late childhood or adolescent, not early childhood (Kimonis et al., 2004; Barry et al., 2008). However, given that CU traits can be detected from early years of life (Willoughby et al., 2011), it may be necessary to explore CU traits and their possible impact on social competence in young children. Second, we know little about the longitudinal association between early CU traits and later social competence. However, considering the findings that CU traits may influence children’s adjustment over long-term (Willoughby et al., 2011; Waller et al., 2016), it may be worthwhile to explore if such effect is evident for children’s social functioning as well. Third, some studies have used relatively small samples (Waschbusch et al., 2007; Barry et al., 2008), or homogeneous samples in terms of race or ethnicity (Barry et al., 2008; Pardini and Fite, 2010). Given that the topic of CU traits in childhood has been raised more recently mostly based on research of Western samples (e.g., Barry et al., 2000; Frick et al., 2015), it may necessary to examine CU traits and its effects in other racial groups such as Asian children. Finally, most previous studies have not considered potential moderators of the association between CU traits and social competence. However, it has been suggested that the effects of CU traits on social competence may in part by determined by other factors such as child executive function (Waller et al., 2017). Thus, in this study, we aim to explore individual and contextual factors that might moderate the association between CU traits and social competence.

### Child Executive Function and Maternal Warmth as Moderators

Inconsistent previous findings raise a possibility that there may be other factors that determine the effects of early CU traits on children’s social competence. In this study, we have chosen to examine the roles of individual (i.e., executive function) and contextual (i.e., maternal warmth) factors as potential moderators of the association between CU traits and social competence. Child executive function and maternal warmth were considered as moderators of the association between early CU traits and social competence because those factors have been found to ameliorate negative effects of early temperamental difficulties on later maladjustment (executive function: Suurland et al., 2016; maternal warmth: Eggum et al., 2009). Such knowledge would be beneficial for explaining complex pathways by which children and their ecology might conjointly determine children’s behavioral outcomes as well as designing efficacious intervention to reduce harmful influence of CU traits.

Executive function refers to a set of abilities that help the child to cognitively and behavioral inhibit automatic responses, and instead think and act deliberately according to contextual demands (Jurado and Rosselli, 2007). Given that inappropriate emotional expression or behaviors leads to interpersonal conflicts and isolation, dysfunctions in executive abilities would be a risk factor of social incompetence. Indeed, children with deficits in executive function are likely to show lower levels of social competence because they experience difficulty regulating their impulses (Jahromi and Stifter, 2008), and also lack foundational skills for social competence such as perspective taking (Carlson et al., 2004). Given that children with high CU traits also show deficits in those critical skills (e.g., low empathy) needed for adequate social functioning (Frick, 1994; Frick et al., 2003), children who demonstrate both high CU traits and low executive function may suffer more problems in relational contexts. Conversely, even if children with high CU display poor affect or empathy, they may not exhibit serious social problems if they are able to use their executive function skills to regulate their impulses. Consistent with this notion, some researchers have proposed that CU traits and executive function may interactively affect children’s social competence. However, there have been few studies on this topic that yielded mixed findings. For example, children with higher levels of CU traits demonstrated more aggressive behavior and were less popular among peers if they also showed lower levels of executive function (Waller et al., 2017). It is possible that children with lower executive function have more difficulty managing their maladaptive behavioral manifestations of CU traits, leading to more problematic social behaviors. Conversely, in another study, adolescents with higher levels with CU traits and early conduct problems demonstrated more violence and substance use if they had higher levels of executive function (Baskin-Sommers et al., 2015). In this study, it was speculated that executive function may enable adolescents to more effectively engage in risky behaviors by helping them contrive craftier strategies to achieve their goals.

In addition to child executive function, maternal warmth was considered as a contextual factor that might affect children’s social competence, both independently and interactively in combination with child CU traits. Parental warmth refers to parents’ emotionally warm and responsive attitudes toward their children (Rohner and Rohner, 1981). Of many parent-related factors, parental warmth has been highlighted as a critical factor for children’s social competence in that it may contribute to positive relational context between the parent and the child, which may promote children’s compliance to parental requests and spontaneous internalization of conduct (Kochanska and Aksan, 1995; Choe et al., 2013). Moreover, parents who demonstrate high levels of warmth toward children are themselves modeling positive behavior that children may utilize in social contexts with peers. Additionally, considering that mothers are primary caregivers for most children, it may be important to examine the role of maternal warmth in shaping social consequences of children who may show early signs of CU traits. Indeed, empirical evidence has supported the association between parental warmth and children’s social competence. For example, higher levels of maternal warmth longitudinally predicted higher levels of empathy and social competence in children (Zhou et al., 2010). In a separate line of research, intervention studies have documented evidence that CU traits may not be fully insensitive environmental influences such as parenting (Kolko et al., 2009; McDonald et al., 2011). If mothers’ warm and responsive parenting can facilitate children with higher levels of CU traits to learn internal attribution and feel guilty about unregulated reactions (Kochanska and Aksan, 2006), then it may be possible to reduce the harmful effects of CU traits on children’s adjustment. Additionally, considering that children with CU traits are more sensitive to rewards than punishment (O’Brien and Frick, 1996), mothers who demonstrate higher levels of warmth may be able to effectively shape and reinforce socially adaptive behaviors. The interactive effect of CU traits and maternal warmth has been investigated in the context of children’s conduct problems (see Waller et al., 2013 for a review). However, no study to date has examined maternal warmth as a moderator of the longitudinal association between CU traits and social competence in young children.

### Consideration of Child Sex

Many of the factors included in this study have been documented to show sex differences. For example, regarding social competence, girls have been found to show higher levels of empathy (Landazabal, 2009), and more prosocial behaviors as well as better problem solving skills in social contexts compared to boys (Walker et al., 2002). Additionally, evidence has also been accumulated that boys show higher levels of CU traits (Viding et al., 2009), and lower levels of executive function (Levin et al., 1991). In addition, mothers with girls have been reported to display higher levels of warmth relative to those with boys (Eisenberg et al., 2001). More importantly, it may be possible that the relations between individual and contextual factors, and children’s social competence differ by child sex. For example, a recent study has found that CU traits were related with girls’ but not boys’ prosocial behaviors (Ciucci et al., 2014). Additionally, effortful control, a construct of self-regulation that mostly overlaps with executive function (Zhou et al., 2012), was more strongly associated with children’s sympathy for boys than girls (Eisenberg et al., 2007). And a lack of maternal warmth was associated with girls’ but not boys’ social incompetence (Chen et al., 1997). Although the literature on the role of child sex on developmental pathways to social competence is small, together the existing findings underscore the need to more actively consider child sex as a factor that might determine those pathways.

### The Present Study

As reviewed, children with CU traits are likely to show insufficient social skills and inappropriate behavioral patterns in social contexts (Frick et al., 2003; Pardini et al., 2003; Pardini and Byrd, 2012). However, there has been relatively little longitudinal research on this topic especially in early childhood. If children’s CU traits can be reliably detected in the early years of life, and if those indices are predictive of children’s future maladaptation, then identifying CU traits in young children may contribute to better informed prevention of problem behaviors before they become more serious and resistant to treatment. More importantly, we know little about individual and contextual factors that might moderate the influence of early CU traits on later social competence. Information on factors that might exacerbate or alleviate the harmful effects of CU traits on later social competence would offer not only theoretical but also clinical benefits by suggesting promising targets for intervention.

Thus, the primary goals of this study were to examine the longitudinal association between CU traits in early childhood and children’s social competence at early school-age, and to test the variations in child executive function and maternal warmth as possible moderators of this relationship. We also considered the possibility that those processes might differ by child sex, and analyzed models separately for boys and girls. Additionally, for more rigorous tests of our models, demographic (i.e., maternal education, Brody and Flor, 1998; family income, Tremblay et al., 2004) and child factors (i.e., language development, Horwitz et al., 2003; externalizing behaviors, Murray-Close et al., 2010) were included as covariates. For the purposes of this study, we used data from a nationally-representative panel study on normally developing Korean children.

Based on prior literature, our hypotheses were as follows: First, children’s CU traits in early childhood would predict lower levels of social competence at early school-age. Second, lower levels of child executive function and lower levels of maternal warmth would be related to lower levels of social competence. Third, child executive function and maternal warmth would moderate the association between CU traits and social competence such that the relationship would be stronger for children who have lower levels of executive function and experience lower levels of maternal warmth. We also explored the possibility that the findings may differ for girls and boys, although we did not generate more specific hypotheses due to a relative lack of relevant prior research.

## Materials and Methods

### Participants

We used longitudinal data from the Panel Study on Korean Children (PSKC) by the Korea Institute of Child Care and Education (KICCE). The PSKC is a prospective cohort study on Korean children’s growth and development. The cohort included 2,078 infants born in 2008 who were selected using stratified multistage sampling. For the purposes of this investigation, PSKC data collected at the 5th (child age 5 years, “Time 1” or “T1” in this study) and 8th (8 years, “Time 2” or “T2”) years were analyzed. A total of 1,004 and 890 families participated at T1 and T2, respectively. Of 890 families who had data at both time points, 643 children had teacher ratings on child social competence available. Thus, the final sample consisted of 643 children (49% girls). At T1, children ranged in age from 48 to 53 months (*M* = 51.04, *SD* = 1.15), and at T2, children were 85–92 months of age (*M* = 87.75, *SD* = 1.45). The racial composition of the sample was homogeneous in that all children in the study were Korean. Mothers’ ages ranged from 24 to 51 years at T1 (*M* = 34.86, *SD* = 3.64), and from 27 years to 54 years at T2 (*M* = 37.97, *SD* = 3.65). The mean family income was $3,983 and $4,283 per month at T1 and T2, respectively. Thirty-five percent of mothers had graduated from a 4-year college, 30% had a 2- or 3-year college degree, and 30% had a high school diploma. The total number of children in the family ranged from 1 to 4 children at T1 (*M* = 2.04, *SD* = 0.63), and from 1 to 5 children at T2 (*M* = 2.22, *SD* = 0.67). At both T1 and T2, families were predominantly first marriage families (98.6% at T1, 97.8% at T2), with smaller numbers of remarried (1.1% at T1, 0.6% at T2), single-parent (0.4% at T1, 1.4% at T2). Results of attrition analyses revealed that families who dropped out of the study at T2 did not differ from those who remained in the study with respect to all study variables except for family income. Specifically, families who did not participate at T2 reported higher income compared to those who stayed in the study (*t* = -3.17, *p* < 0.01).

### Measures

#### Child CU Traits

Five items on the Child Behavior Checklist (CBCL) for ages 1½–5 (Achenbach and Rescorla, 2000) were selected to measure behavioral manifestations of CU traits in early childhood (i.e., “doesn’t seem to feel guilty after misbehaving,” “punishment doesn’t change behavior,” “seems unresponsive to affection,” “shows little affection toward people,” “shows too little fear of getting hurt”). At 5 years of child age, mothers rated each item on a 3-point scale (0 = *not true*, 2 = *very true* or *often true*). Prior efforts to gauge CU in children as young as 3 years have documented that those items may represent behaviors that are empirically distinct from ADHD or ODD (Willoughby et al., 2011), but uniquely related to major aspects of CU such as moral dysregulation, and a lack of empathy and guilt. Internal reliability for the CU factor in this study was not high (α = 0.53), which was also consistent with earlier studies using the same set of items (α = 0.55, Willoughby et al., 2014; Waller et al., 2017).

#### Child Executive Function

At 8 years, child executive function was measured using the Child-Adolescent Self-reported Executive Function Difficulty Screening Questionnaire (Song, 2014b) that consists of four subscales to assess deficits in executive function: planning-organizing difficulty (11 items; e.g., “has difficulty in setting a goal and making real actions to achieve it”), behavior control difficulty (11 items; e.g., “has more difficulty in regulating one’s own behavior compared to other children of the same age”), emotional control difficulty (8 items; e.g., “overreacts to trifles”), attention-concentration difficulty (10 items; e.g., “often loses one’s belongings or homework”). This scale was developed based on two existing scales of executive function (Gioia et al., 2000; Grace and Malloy, 2001), each of which have been reported to demonstrate significant correlations with lab tasks of executive function such as the Trail-making Test (Velligan et al., 2002; Toplak et al., 2008), subtests of the Wechsler Intelligence Scale for Children (WISC) that assess working memory, the stocking of Cambridge task (Toplak et al., 2008), and Verbal Fluency (Velligan et al., 2002). Although the scale was originally developed as a self-report questionnaire, in this study, mothers completed it based on their observation of their children’s behavior. Each item was rated on a 3-point scale (1 = *never true*, 3 = *often true*), and higher scores represented higher deficits in a child’s executive function abilities (α = 0.94). With respect to the Korean population, this scale has been found to correlate with the Stroop Test (Song, 2014b). The scale was also found to be useful in predicting child outcomes that are known to be associated with executive function (e.g., academic achievement, Song, 2014a).

#### Maternal Warmth

At 5 years, mothers completed the Korean Parenting Style questionnaire that consists of two subscales of warmth and control (Cho et al., 1999). For the purposes of this study, six items on the warmth factor were analyzed (e.g., “has intimate time with a child”). Each item was rated on a 5-point scale (1 = *never true*, 5 = *very true*), and higher scores represented higher maternal warmth toward their child (α = 0.87).

#### Child Social Competence

At 8 years, teachers rated children’s behavior on the School Adjustment Inventory for 1st grade Elementary School Students (Chi and Jung, 2006). This scale is composed of four subscales including adjustment in school life (11 items), adjustment in academic performance (11 items), adjustment in peer relationship (8 items), adjustment in the relation with teacher (5 items). Each item was measured on a 5-point scale (1 = *never true*, 5 = *very true*). In this study, social competence was assessed using scores on the adjustment in peer relationship subscale that included items that tap evaluated a child’s prosocial behaviors and problem solving skills in peer contexts (e.g., “helps friends well,” “consoles a friend when he/she is sad,” “resolves conflicts with friends in positive ways”). Higher scores indicated more advanced social competence in children (α = 0.95).

#### Covariates

Based on previous research, individual (language development, externalizing behavior) and family demographic (maternal education, family income) factors were included in the analysis to control for their potential effects on children’s social competence. At 5 years, children’s language development was rated by teachers using the Korean Evidence-Based Assessment for Young Children (Lee et al., 2008) that includes 11 items (0 = *no*, 1 = *yes*) that measure a child’s receptive and expressive language skills (α = 0.51). At 5 years, the broadband Externalizing scale on the CBCL 1½–5 (Achenbach and Rescorla, 2000) was used to assess variations in children’s externalizing behavior (α = 0.87). It also should be noted that two items on the Externalizing scale that overlapped with CU (i.e., “doesn’t seem to feel guilty after misbehaving,” “punishment doesn’t change behavior”) were excluded. Maternal education was rated on an 8-point scale (1 = *no formal education*, 8 = *doctoral degree*), and family income was reported by mothers as average monthly income for the family.

### Analysis Plan

Following preliminary analyses (i.e., descriptive statistics, *t*-tests by child sex, bivariate correlations), hierarchical regression models were analyzed to examine the longitudinal effects of child CU traits at 5 years on social competence at 8 years, and to test if this association might be moderated by child executive function or maternal warmth, and child sex. Analysis was performed separately for each moderator using SPSS Statistics 20. Specifically, in Step 1, covariates were included (i.e., maternal education, family income, child language ability, and child externalizing behavior). In Step 2, child CU traits and either child executive function or maternal warmth were entered to examine their main effects on child social competence. In Step 3, a two-way interaction term of child CU × child executive function or child CU × maternal warmth was included. Finally, in step 4, a three-way interaction between child CU × child executive function (or maternal warmth) × child sex was analyzed. In those processes, variables were centered to minimize multicollinearity except child sex (Aiken et al., 1991), and significant interaction effects were probed using simple slopes procedures (Aiken et al., 1991).

## Results

### Preliminary Analyses

Descriptive statistics and independent *t*-tests by child sex for all study variables are presented in Table 1. Except for maternal education, sex differences were significant such that girls showed more developed language abilities, executive function, and social competence than boys, whereas boys demonstrated higher levels of CU traits and externalizing behavior. Girls’ mothers reported higher levels of warmth toward their children than those of boys, and families with boys had lower family income than those with girls. Bivariate correlations also revealed different results by child sex (Table 2). For boys, social competence was significantly related to more advanced language abilities, lower CU traits, less deficits in executive function, and fewer externalizing behavior. For girls, social competence was correlated with higher scores in language abilities, but was not significantly associated with CU, executive function, or externalizing behavior. For both girls and boys, maternal warmth was not a significant correlate of variability in children’s social competence.

**Table 1:** Descriptive statistics and independent *t*-tests by child sex (*N* = 643).

	Boys (*N* = 328)		Girls (*N* = 315)		*t*
	*M*	*SD*	*M*	*SD*	
Maternal education	5.17	1.06	5.22	1.16	–0.573
Family income	400.03	245.31	451.14	387.94	–2.00*
Child externalizing behaviors at age 5	7.82	5.43	6.81	5.14	2.38*
Child language development at age 5	104.40	12.42	107.36	9.65	–3.37**
Child CU traits at age 5	1.21	1.30	0.99	1.20	2.20*
Child executive function deficits at age 8	59.59	12.44	54.81	11.01	5.16***
Maternal warmth at age 5	21.79	3.39	22.37	3.17	–2.26*
Child social competence at age 8	30.18	6.88	33.00	6.07	–5.49***


*^∗^*p* < 0.05, ^∗∗^*p* < 0.01, ^∗∗∗^*p* < 0.001.*

**Table 2:** Bivariate correlations of study variables by child sex (*N* = 643).

	1	2	3	4	5	6	7	8
1. Maternal education	–	0.15**	0.02	–0.07	–0.03	–0.09	0.11*	0.08
2. Family income	0.23**	–	0.50	–0.13*	–0.08	–0.06	0.04	0.06
3. Child language development at age 5	0.01	–0.14**	–	–0.09	0.06	–0.07	0.00	0.15**
4. Child externalizing behaviors at age 5	–0.10	–0.08	0.06	–	0.55**	0.46**	–0.23**	–0.10
5. Child CU traits at age 5	–0.09	–0.05	0.03	0.68**	–	0.32**	–0.16**	–0.02
6. Child executive function deficits at age 8	–0.06	0.00	–0.03	0.40**	0.31**	–	–0.18**	–0.06
7. Maternal warmth at age 5	0.07	0.04	0.06	–0.27**	–0.28**	–0.32**	–	–0.01
8. Social competence at age 8	0.11*	–0.03	0.13*	–0.16**	–0.21**	–0.22**	0.08	–


*Numbers appearing below and above the diagonal are for boys and girls, respectively. ^∗^p < 0.05, ^∗∗^p < 0.01.*

### Hierarchical Regression Models

**FIGURE 1:**
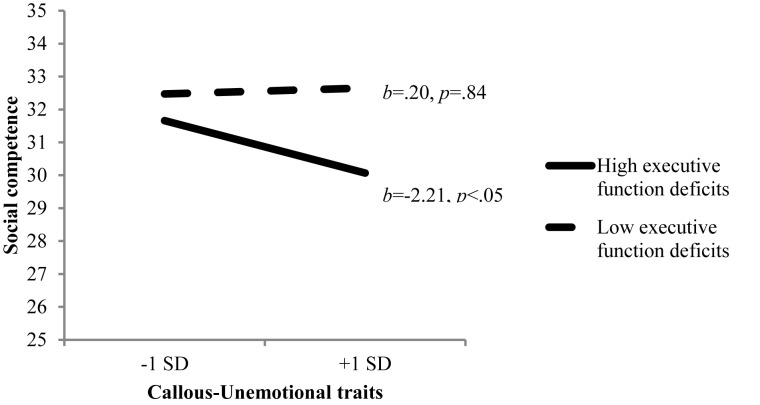
CU traits × executive function deficits predicting social competence.

Results of a hierarchical regression model to examine additive and interactive effects of CU traits at 5 years, executive function at 8 years, and child sex on children’s social competence are presented in Table 3. Controlling for the effects of covariates, child executive function at 8 years, *β* = -0.27, *p* < 0.05, and child sex, *β* = 0.16, *p* < 0.05, demonstrated significant main effects on social competence at 8 years such that children with lower executive function or boys were rated by teachers to be less socially competent. A two-way interaction of CU traits and executive function was not significant, *β* = -0.24, *p* = 0.07, but we probed the interaction using a simple slopes procedure as the result demonstrated a trend. As presented in Figure 1, CU traits were negatively associated with social competence for children with more problems in executive function, *b* = -2.21, *p* < 0.05, but not for children with less deficits in executive function, *b* = 0.20, *p* = 0.84. Finally, the three-way interaction of CU traits × executive function deficits × child sex was non-significant. This model accounted for 12% of variability in children’s social competence. Results of the same regression model without covariates were almost identical in terms of the predictors and magnitude of statistical significance, except that, without covariates, the interactive effect of CU traits × executive function was significant, *β* = -0.27, *p* < 0.05.

**Table 3:** Additive and interactive effects of CU traits, executive function deficits, and child sex on children’s social competence (*N* = 643).

Variables	Outcome variable: social competence at age 8 years		
	B (SE)	β	R^2^ (ΔR^2^)
**STEP 1**			
Maternal education	0.46 (0.23)	0.08^∗^	0.055^∗∗∗^
Family income	0.00 (0.00)	0.01	
Child language development at age 5	0.07 (0.02)	0.13^∗∗^	
Child externalizing behaviors at age 5	–0.04 (0.07)	–0.03	
**STEP 2**			
Child CU traits at age 5	–0.98 (0.74)	–0.19	0.101^∗∗∗^
Child executive function deficits at age 8	–0.15 (0.07)	–0.27^∗^	(0.046^∗∗∗^)
Child sex	1.48 (0.70)	0.11^∗^	
**STEP 3**			
CU traits × executive function deficits	–0.10 (0.05)	–0.24^†^	0.117^∗∗∗^
CU traits × child sex	0.52 (0.45)	0.15	(0.016^∗^)
Executive function deficits × child sex	0.06 (0.05)	0.18	
**STEP 4**			
CU traits × executive function deficits × child sex	0.05 (0.03)	0.18	0.120^∗∗∗^
			(0.003)


*For child sex, 1 = boys, 2 = girls. ^†^p < 0.10, ^∗^p < 0.05, ^∗∗^p < 0.01, ^∗∗∗^p < 0.001.*

**FIGURE 2:**
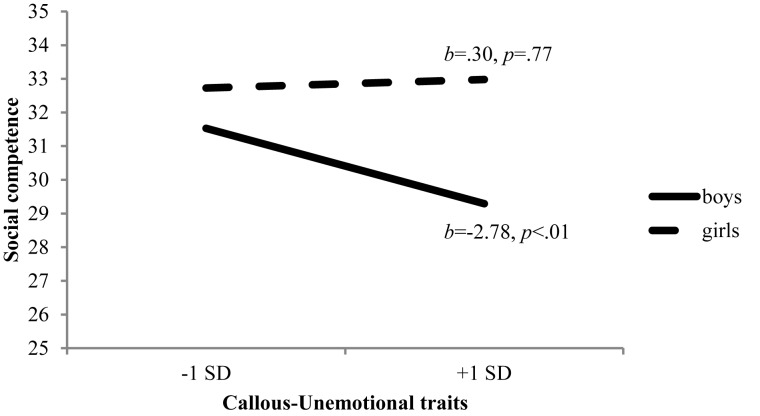
CU traits × child sex predicting social competence.

Subsequently, additive and interactive effects of CU traits at 5 years, maternal warmth at 5 years, and child sex on children’s social competence at 8 years were tested in a hierarchical regression (Table 4). Controlling for the effects of covariates, CU traits showed significant main effects on social competence, *β* = -0.32, *p* < 0.05, such that higher levels of CU traits were predictive of lower levels of social competence 3 years later. A two-way interaction of CU traits and child sex was also significant, *β* = 0.26, *p* < 0.01. Probing this interaction using a simple slope procedure (Figure 2), it was found that early CU traits were negatively associated with later social competence for boys, *b* = -2.78, *p* < 0.01. Conversely, the association between CU traits and social competence was not significant for girls, *b* = 0.30, *p* = 0.77. Finally, no effects involving maternal warmth, both main effects and interactive effects, were statistically significant. This model accounted for 10% of variability in children’s social competence. Results of the same regression model without covariates were almost identical in terms of the predictors and magnitude of statistical significance.

**Table 4:** Additive and interactive effects of CU traits, maternal warmth, and child sex on children’s social competence (*N* = 643).

Variables	Outcome variable: social competence at age 8 years		
	B (SE)	β	R^2^ (ΔR^2^)
**STEP 1**			
Maternal education	0.48 (0.23)	0.08^∗^	0.06^∗∗∗^
Family income	0.00 (0.00)	0.00	
Child language development at age 5	0.08 (0.02)	0.14^∗∗∗^	
Child externalizing behaviors at age 5	–0.08 (0.06)	–0.06	
**STEP 2**			
Child CU traits at age 5	–1.69 (0.70)	–0.32^∗^	0.092^∗∗∗^
Maternal warmth at age 5	0.02 (0.25)	0.01	(0.038^∗∗∗^)
Child sex	1.42 (3.75)	0.11	
**STEP 3**			
CU traits × maternal warmth	–0.03 (0.19)	–0.02	0.108^∗∗∗^
CU traits × child sex	0.90 (0.43)	0.26^∗^	(0.015^∗^)
Maternal warmth × child sex	0.00 (0.16)	0.00	
**STEP 4**			
CU traits × maternal warmth × child sex	0.12 (0.13)	0.11	0.109^∗∗∗^
			(0.001)


*For child sex, 1 = boys, 2 = girls. ^∗^p < 0.05, ^∗∗∗^p < 0.001.*

## Discussion

The goals of this study were to examine the longitudinal effect of children’s CU traits in early childhood on their later social competence in school and to test whether child executive function and maternal warmth might moderate the relationship. Results indicated that, in the model with executive function as a moderator, higher levels of deficits in child executive function and being boys had significant negative effects on children’s social competence. However, the association between early CU traits and executive function only demonstrated a trend indicating that the association between CU and social competence was only significant for children with higher deficits in executive function. Additionally, in the model with maternal warmth as a moderator, higher levels of child CU traits at 5 years was predictive of lower levels of social competence at 8 years, and this effect was more pronounced for boys as indicated by a significant two-way interaction of CU traits × child sex on children’s social competence. The findings were significant after controlling for variations in maternal education and family income as well as children’s language abilities and externalizing behavior.

### Early CU Traits as a Predictor of Social Competence

This study represents an initial attempt to create a construct of CU traits within a sample of Korean preschoolers. Although it has been proposed that CU traits may appear from very early age (Dadds et al., 2005; Waller et al., 2012), previous studies have typically focused on middle childhood or early adolescence (Pardini et al., 2003; Kimonis et al., 2004; Barry et al., 2008). Our findings add to the literature by providing evidence that early CU traits may be captured using the same items as earlier Western studies on this topic (e.g., Willoughby et al., 2011; Waller et al., 2017). Although the reliability was not impressive, which is a remaining task for the future, this was consistent with earlier investigations as well.

The finding that early CU traits predicted children’s social competence at early school-age is in concert with previous studies on this topic (Waschbusch and Willoughby, 2008; Waller et al., 2017; Haas et al., 2018). It is speculated that young children who show CU behaviors such as deficits in sympathizing with others’ feelings and insensitivity to negative consequences may experience difficulty acquiring and modifying their behavior in interpersonal contexts. Interestingly, this study suggests sex-differentiated pathways to children’s social competence, which has been rarely explored in prior research. Earlier studies that have incorporated child sex have typically treated it as a covariate to statically control for its effects on their models rather than to examine how the findings may differ by child sex (Waschbusch and Willoughby, 2008; Waller et al., 2017). However, we tested differential pathways by child sex, and found that the longitudinal relationship between CU traits and social competence more pronounced for boys. Although we did not have data to explore reasons why CU traits may serve as a more salient risk factor for boys, there may be several possibilities. For example, early pathways by which boys and girls develop social competence may not be the same, involving different risk and protective factors. In fact, the possibility of differential risk mechanisms for girls and boys have been proposed in the literature on child externalizing based on the findings of different individual and contextual factors for boys’ versus girls’ behavior problems (Hill et al., 2006; Chang et al., 2011). However, this does not mean that girls’ CU traits do not pose a risk for their adjustment as indicated by significant main effects of CU on girls’ social competence. Nevertheless, more research is needed to better understand gender differences in the effects of CU traits on children’s functioning, incorporating multiple developmental periods and other maladaptive behaviors that were not examined in this study.

Furthermore, the discrepant finding for boys and girls in this study may be due to the fact that scores of CU traits and social competence demonstrated significant sex differences favoring girls. The distribution of CU scores was more restricted for girls, which may have resulted in a smaller finding for girls regarding the relationship between CU traits and social competence. To complicate the matter even further, mothers and teachers may have considered, albeit implicitly, the sex of the child when rating their behaviors. For example, girls who had high scores in maternal reports of CU traits may not demonstrate similar behaviors to boys who had the same scores. Teachers may have perceived boys’ and girls’ similar social behaviors differently leading to different ratings. Although both CU traits and social behavior have been documented to show sex differences (Walker et al., 2002; Viding et al., 2009), it would be necessary to confirm that discrepant findings by child sex in this study indeed reflects sex-differentiated pathways rather than sample characteristic or reporter subjectivity by replicating the study within a high risk sample using more diverse methodology to cross-validate different measures of child behavior.

### Child Executive Function and Maternal Warmth as Moderators

A unique feature of this study was our consideration of individual (i.e., child executive function) and contextual (i.e., maternal warmth) factors that may shift pathways by which early CU traits become associated with later social competence in children. Disappointingly, the moderating effects of child executive function or maternal warmth were all non-significant. However, the findings were slightly different for the two moderators considered in this study. Specifically, there was a trend in the interactive effects of CU traits and child executive function on later social competence such that higher CU traits predicted lower social competence only for children who had lower scores in executive function. This is consistent with prior findings that children who show both higher levels of CU traits and deficits in executive function suffer from higher levels of peer rejection (Waller et al., 2017). In this study, deficits in executive function had significant main effects on children’s social competence as well. As children with lower levels of executive function abilities may have more difficulty suppressing inappropriate responses (Miyake et al., 2000) which is a risk factor for aggressive and impulsive behaviors (Williams et al., 2009), they may be viewed as less competent in social contexts. Further, given that they are unlikely to display adequate social skills like perspective taking (Carlson et al., 2004), the result can be also interpreted as the effect of apathetic and blunted interpersonal style.

Additionally, children who are better able to regulate their cognitive and behavioral impulses (i.e., good executive function abilities) may be able to refrain from automatically enacting upon their CU tendencies with socially undesirable behavior in social contexts. Thus, the finding that the negative association between CU traits and social competence might exist only for children with lower levels of executive function suggest that executive function may mitigate negative impact of early CU by helping them to regulate their social behavior according to contextual demands.

Similar to child executive function, maternal warmth that was hypothesized as a contextual factor that might strengthen or weaken the effects of CU traits on children’s social competence did not yield significant findings. However, this is consistent with some treatment studies that included parenting intervention did not find significant effect on social competence of children with CU traits (Hawes and Dadds, 2005; White et al., 2013), suggesting a possibility that maternal warmth may not moderate the association between children’s CU traits and social competence. Furthermore, at least in relation to parental warmth, this finding may support a previous suggestion that CU traits tend to be insensitive to environmental influences (Wootton et al., 1997; Oxford et al., 2003). It is also possible that unique features of Korean families might have contributed to the current findings. For example, in Asian countries that can be grouped as Confucian cultures, children are strongly encouraged to control their needs and impulses to promote harmonious relationship with others (Ho, 1986; Kwon, 2002), facilitating the development of self-regulatory abilities from an early age. Indeed, empirical studies have documented that Asian children may demonstrate higher executive function (Zhou et al., 2012) than those of western cultures (Sabbagh et al., 2006; Lewis et al., 2009). In such a context, children with deficits in executive function may be much more visible and at risk for unoptimal parenting behavior. Additionally, it is possible that Korean mothers may display their affection differently from their Western counterparts. For example, there has been a suggestion that Korean mothers are less likely to display their affection to their children in forms of kissing, hugging, or praising (Kim and Hong, 2007). However, due to the lack of theory and empirical studies on cultural influences on parental attitude and their impact on child adjustment, it is different at this point to speculate how possibly different manifestations of maternal warmth may have common versus unique role on the pathway by which early CU becomes associated with child outcomes. Thus, to more fully understand complex processes that underlie the pathways by which early CU traits, child executive function, and maternal warmth may together shape children’s social competence, it is necessary to incorporate other aspects of parenting as well as other methods of assessment.

### Limitations, Future Directions, and Conclusions

There are a few limitations to note. First, participants were part of a panel study of Korean children, and thus generalizing our findings to a clinical population or other ethnic groups might be limited. In particular, CU traits are known to be quite rare in the community sample (Herpers et al., 2012), replicating the current study in high risk samples is needed (e.g., children with conduct disorder). Additionally, attrition analyses revealed that higher income families had higher drop-out rates. Although the reasons for the differential drop-out is unknown, this may also limit generalizability of the current findings as family’s socioeconomic status is a well-established factor that impacts children’s adjustment.

Second, our measure of CU traits (i.e., 5 items on CBCL) was not originally developed to assess CU characteristics in children. As children may demonstrate CU behaviors that may differ from those of adolescents, we have chosen to use items from a more comprehensive behavior checklist rather than to use existing measures of CU traits for adolescents (Frick and Hare, 2001; Essau et al., 2006). Although our decision was based on earlier studies that have provided empirical evidence that those items may be valid indicators of young children’s CU (e.g., Willoughby et al., 2011), it should be noted that CBCL was not designed to measure children’s stable personality traits such as CU. In addition, the internal consistency of our CU measure was low, indicating that this index may be heterogeneous in its composition. However, inter-item correlations demonstrated that no particular item(s) were driving this problem. The low reliability of CU may be due to the possibility that, at early ages, behavioral manifestations of CU may not only reflect underlying CU traits (e.g., lack of empathy), but also egocentrism that may be normative at this developmental period (Hoffman, 2001). Thus the CU composite in this study may represent a combination of both genuine CU traits and seemingly CU-like behaviors that children may outgrow over time, and this might be resulted in a relatively low reliability of this measure. Hence, future research may benefit by formally developing a measure to reliably assess behavioral manifestations of children’s CU traits.

Third, because we analyzed cohort data, measurements of variables were not satisfactory. For example, although using teacher ratings for social competence was a strength of this study, all other factors relied on maternal reports which may have led to an overestimation of relations between variables. Additionally, the two moderators were assessed at different time points (child executive function at 8 years, maternal warmth at 5 years), and thus it is not possible to rule out the possibility that their effects on the association between CU traits and social competence may in fact be reflective of diverging processes involved at different developmental periods.

Finally, although we used longitudinal data and controlled for the influence of intrachild factors (i.e., language, externalizing) on children’s social competence, it is impossible to draw conclusions on causality or directionality of the effects examined in this study. The majority of individual and contextual factors included in our model are likely to be interrelated in complex ways (e.g., bidirectional effects between child and parent factors) which were not possible to disentangle using the current dataset. Future research might benefit by tracking variables over time and considering their stability and change in more ecologically valid model to reveal early pathways to children’s social competence.

In summary, this study represents an early effort to investigate the longitudinal association of CU traits in the preschool period and children’s social competence at early school-age. More importantly, our results suggest that those effects might be marginally influenced by variations in child executive function as a moderator, and that child sex needs to be considered as a determinant of early pathways to social competence. If replicated, the findings reveal a complex interplay of individual and contextual factors that lead to individual differences in children’s ability to navigate the social world, and inform clinical efforts for early identification and prevention of problem behavior in relational contexts.

## Author Contributions

HK was responsible for literature review, data analysis, and write-up. HC advised all parts of the study and write-up.

## Conflict of Interest Statement

The authors declare that the research was conducted in the absence of any commercial or financial relationships that could be construed as a potential conflict of interest.
